# Antitumor response to microscopic melanoma in the gastric mucosa mimicking ipilimumab-induced gastritis

**DOI:** 10.1186/s40425-019-0524-1

**Published:** 2019-02-11

**Authors:** Elisa Bello, Justine V. Cohen, Mari Mino-Kenudson, Michael Dougan

**Affiliations:** 1 Division of Gastroenterology, Department of Medicine, Massachusetts General Hospital, Boston, MA USA; 2 Division of Oncology, Department of Medicine, Massachusetts General Hospital, Boston, MA USA; 3 Harvard Medical School, Boston, MA USA; 4 Department of Pathology, Massachusetts General Hospital, Boston, MA USA

## Abstract

**Background:**

Alongside its clinical success, checkpoint blockade has also given rise to a set of immune-related adverse events (irAEs). In addition to causing considerable morbidity and even mortality, irAEs may limit the success and scope of immunotherapy. Most irAEs arise at mucosal barriers, including the gastrointestinal mucosa, leading most commonly to colitis, though both gastritis and enteritis can result from checkpoint blockade. While guidelines generally recommend confirmatory testing for suspected severe irAEs, the role of endoscopy in diagnosing more moderate irAEs is less clear. Many patients with suspected gastrointestinal irAEs are treated empirically with glucocorticoids based on typical symptoms. Although efficient, this approach may miss less common underlying etiologies, and may expose patients unnecessarily to an increased risk of infection, and a potentially dampened antitumor response.

**Case presentation:**

We report a case of ipilimumab-induced antitumor immunity targeting microscopic gastric melanoma metastases, mimicking checkpoint blockade induced gastritis. Immune suppression was avoided and the immunotherapy was continued.

**Conclusion:**

Checkpoint blockade can induce rapid inflammatory responses to tumor tissue present throughout the body. These responses are desirable, but may also lead to local tissue injury, causing symptoms that may mimic adverse events. This is particularly important to consider in organs where metastatic disease may be unappreciated at the time of treatment, and where irAEs are otherwise common, such as the gastrointestinal tract. In this setting, empiric immune suppression may inhibit antitumor responses, improving symptoms but at a potential cost to therapeutic efficacy.

## Background

Monoclonal antibodies that block the immune checkpoint receptors CTLA-4, PD-1, and PD-L1 are now standard of care for a wide range of malignancies [1–3]. Despite the significant survival advantage conferred by these immunotherapies, they have also given rise to a new subset of immune-related adverse events (irAEs) that resemble sporadic autoimmune diseases, such as ulcerative colitis or rheumatoid arthritis [4–6]. These immune toxicities relate to the endogenous function of the checkpoint receptors which is to suppress auto-inflammatory responses [4–6]. In addition to causing considerable morbidity and even mortality, these inflammatory side effects may limit the success and scope of immunotherapy, particularly in the setting of combination treatments [4–7].

Most checkpoint blockade induced toxicities arise at mucosal barriers such as the lung, gastrointestinal (GI) mucosa, and skin [4–6]. These organs serve as an interface with the outside world where distinguishing between dangerous invading organisms and normal commensal flora is of critical importance. In general, irAEs respond to local or systemic glucocorticoids, which are often given empirically [4]. While guidelines generally recommend testing in the setting of severe toxicity, the role of diagnostic testing, such as endoscopy, in the diagnosis of checkpoint blockade induced irAEs remains poorly studied [4, 5, 8–10]. Endoscopic evaluation has an important role in the diagnosis and monitoring of multiple GI pathologies, often directly indicating specific treatments [4]. We present the case of a patient with metastatic uveal melanoma treated with sequential pembrolizumab (anti-PD-1) followed by ipilimumab (anti-CTLA-4) who developed sudden onset reflux and decreased appetite shortly after starting ipilimumab. Biopsy revealed microscopic melanoma infiltrating the gastric mucosa and provoking a local inflammatory response resembling gastritis. These findings suggest that the patient’s inflammatory symptoms were not side effects of checkpoint blockade but rather were the inflammatory consequence of effective antitumor immunity.

## Case presentation

Ms. C is a 73-year-old woman diagnosed with uveal melanoma in 2014 and initially treated with proton beam radiation therapy. Magnetic resonance imaging (MRI) conducted in November 2015 as part of disease surveillance confirmed liver metastases. The patient’s past medical history included angiomyolipoma of the kidney, uterine leiomyoma, obstructive sleep apnea, and enthesopathy in the hip, Achilles tendinitis, and arthritis, and she had been previously treated with a bone graft. Her medications were notable for estradiol-norethindrone, and trazodone. She had allergies to gabapentin, and had no family history of inflammatory bowel disease or GI malignancy.

**Fig. 1:**
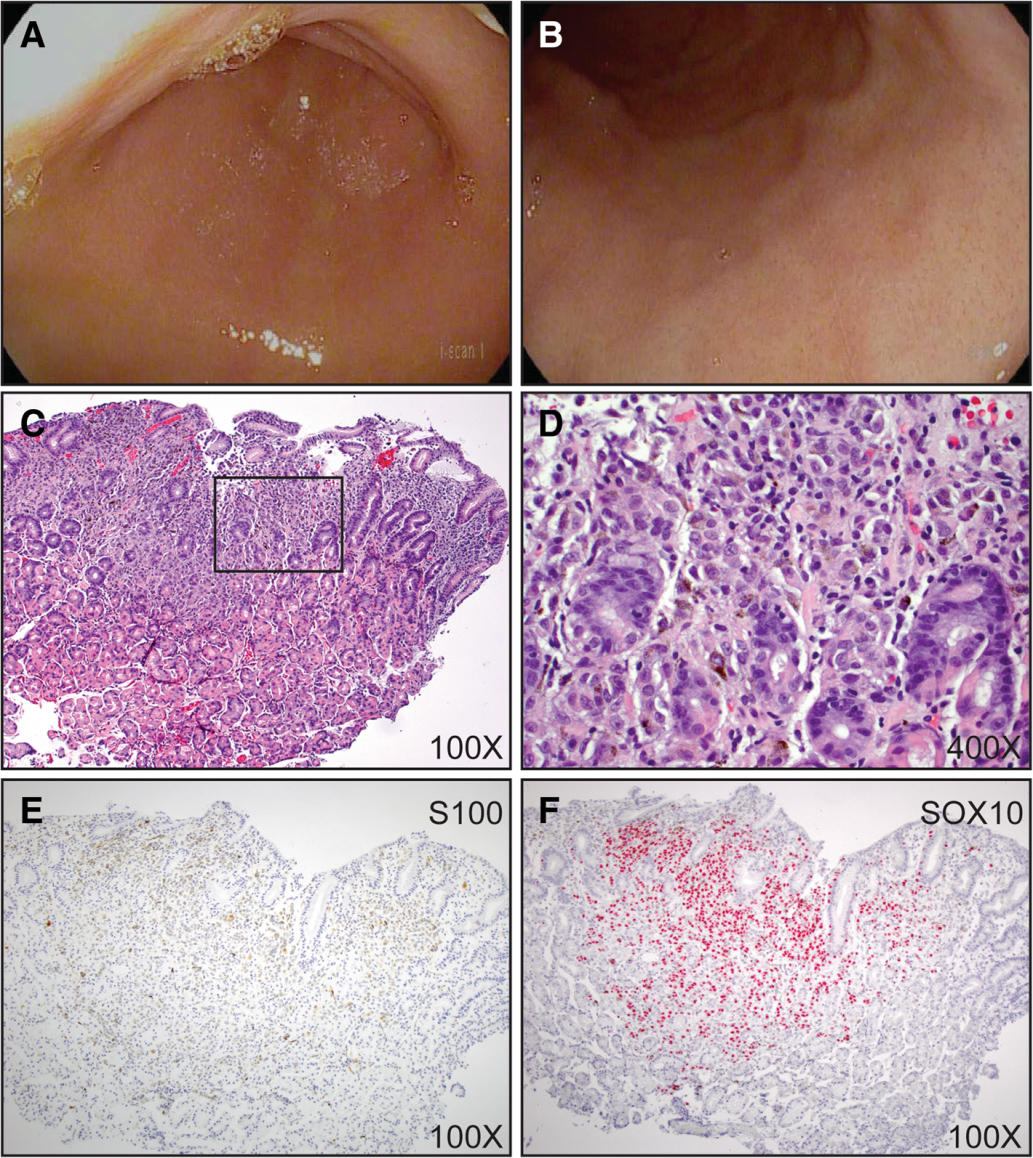
Gastritis from ipilimumab-associated antitumor response in the gastric mucosa. **a** and **b** endoscopic photographs of the gastric antrum (**a**) and fundus (**b**). 100X (**c**) and 400X (**d**) magnification images of hematoxylin and eosin stain of biopsies from the gastric mucosa. **e** S100 and **f** SOX10 immunohistochemistry performed on biopsies from the gastric mucosa

Her liver metastases were initially treated with pembrolizumab every 3 weeks beginning in December 2015. Selective internal radiation therapy (SIRT) was performed via the right hepatic artery. In April, 2016, after the 5th cycle of pembrolizumab, positron emission tomography computed tomography (PET-CT) scans found new pulmonary metastases. Pembrolizumab was discontinued for progression, and she began ipilimumab 3 mg/kg as second line immunotherapy on April 29th. One day prior to starting ipilimumab, she was seen in the emergency department with new onset paroxysmal atrial fibrillation for which she was started on metoprolol and apixiban. Following her second dose of ipilimumab, she developed epigastric pain and symptoms of gastric reflux, both of which were unresponsive to high dose proton pump inhibitors (pantoprazole 40 mg twice daily) and Carafate. She also had new onset, mild diarrhea with 3–4 loose stools daily. Esophagogastroduodenoscopy and flexible sigmoidoscopy were performed to inform further treatment. The esophageal, gastric and duodenal mucosa appeared normal on endoscopic examination without evidence of ulceration, or other significant mucosal injury (Fig. 1 a and b). Gastric biopsies demonstrated lymphocytic inflammation surrounding pigmented cells scattered throughout the gastric mucosa (Fig. 1. c and d). S100 and SOX10 immunostains confirmed multiple microscopic foci of melanoma with tumor associated inflammation resembling gastritis (Fig. 1. e and f). Flexible sigmoidoscopy and colonic biopsies were normal.

Glucocorticoid treatment was initially deferred as this was considered an appropriate antitumor response, and she received her third dose of ipilimumab. After the 3rd dose, she developed an elevation in her transaminases and bilirubin concerning for checkpoint hepatitis. She was started on glucocorticoids and further immunotherapy was held. Her hepatitis resolved on glucocorticoids. Her gastric symptoms also improved on glucocorticoid therapy consistent with an immune-mediated mechanism. The patient ultimately died from complications of her melanoma six months after starting ipilimumab.

## Conclusion

Immunotherapy prolongs survival across a diverse group of cancers, but in a minority of patients, these treatments also lead to important inflammatory toxicities that are not seen with conventional chemotherapy or other targeted agents [1–7]. In the setting of severe irAEs, immunotherapy is often delayed or discontinued [4, 5, 8, 9]. Systemic glucocorticoids are currently the cornerstone of management for most irAEs, but the effect of this broad immunosuppression on antitumor immunity is not well understood [4, 5, 8, 9, 11, 12].

The appropriate balance between empiric symptom-based treatment with glucocorticoids, and management based on tissue diagnosis is not well studied, and is likely to differ based on the specific toxicity, and even the underlying malignancy. Toxicities involving the gastrointestinal mucosa are among the most commonly seen for both CTLA-4 blockade and that of PD-1/PD-L1, although the severity of CTLA-4 blockade GI toxicities is considerably higher than for that of PD-1/PD-L1 [4]. While treatment associated GI symptoms including diarrhea, urgency, nausea, vomiting, and decreased appetite often represent inflammatory toxicities from immunotherapy, these symptoms poorly correlate with the severity of mucosal inflammation, and a more thorough exclusion of alternative diagnoses may be appropriate in many patients with more severe symptoms [4, 10, 13, 14]. Empiric glucocorticoid-treatment is not without risks, including the possibility that immunosuppression may limit the scope of effective antitumor responses [11]. Current society guidelines based on expert opinion suggest endoscopic evaluation for patients with grade ≥ 2 symptoms, though less consensus exists on whether this should be done prior to initiation of corticosteroids [5, 8, 9]. The role of upper endoscopy is also incompletely explored in present guidelines, largely due to inadequate published data on this topic.

The pathophysiology of inflammation in the GI tract induced by checkpoint blockade remains poorly understood [4]. Biopsies confirm the presence of dense T cell infiltrates in keeping with the known mechanism of action of the checkpoint blockade, and response to TNF-α blockade suggests a critical role for this cytokine in mediating this inflammatory toxicity [4]. Furthermore, successful treatment of checkpoint colitis using blockade of the gut homing integrin α4β7 suggests that migration of T cells from the blood into the colonic mucosa plays an important role in continuing this inflammatory response [15].

Melanoma is known to metastasize to the GI mucosa, although most patients with metastatic disease do not have GI mucosal involvement [16]. Furthermore, gastric mucosal involvement is a particularly unusual sight of metastasis in uveal melanoma. In this case, our evaluation uncovered unexpected microscopic gastric metastases that appeared to be actively targeted by the immune system following initiation of ipilimumab. The patient’s symptoms were thus a consequence of appropriate antitumor immunity rather than an inflammatory toxicity. Appropriate diagnosis enabled the patient to receive additional immunotherapy and delay systemic glucocorticoids, though after a 3rd dose of ipilimumab she developed checkpoint hepatitis. Treatment of her hepatitis with glucocorticoids led to concomitant improvement in her GI symptoms, demonstrating that her symptoms were likely inflammatory in nature, though not a classic irAE. The frequency with which suspected irAEs are excluded by endoscopic evaluation is currently unclear, though a retrospective analysis of 182 patients evaluated at MD Anderson Cancer Center by lower endoscopy found that 129 had histologically confirmed inflammation (71%) [10]. This is similar to our own experience, where 63 (66%) patients with melanoma out of 96 evaluated endoscopically had biopsy confirmed inflammation.

This case underscores the complexity of caring for patients on immunotherapy who develop treatment-associated symptoms. While most irAEs occur in a predictable manner and respond to empiric treatment, a substantial minority of patients present with non classic symptoms, or with typical symptoms arising from an unusual etiology, as is underscored by the case presented here [4–6, 17]. Empiric glucocorticoid treatment is clearly not without risk, exposing patients to the risk of infection, and having the potential to reduce antitumor responses [4, 11, 17]. Determining the optimal threshold for detailed diagnostic evaluations is an important goal, and one that has not been adequately studied. As we develop future guidelines for irAE management, careful attention will have to be paid to the role of rare presentations in diagnostic and management algorithms. This will take a multidisciplinary approach, and is likely to become increasingly clinically important as more diverse immunotherapy combinations enter the clinic.
